# Effect of Alkyl Chain Length on Carboxylic Acid SAMs on Ti-6Al-4V

**DOI:** 10.3390/ma5071206

**Published:** 2012-07-09

**Authors:** Gavin A. Buckholtz, Ellen S. Gawalt

**Affiliations:** 1 Department of Chemistry and Biochemistry, Duquesne University, 600 Forbes Avenue, Pittsburgh, PA 15282, USA; E-Mail: buckholtzg@duq.edu; 2 McGowan Institute for Regenerative Medicine, University of Pittsburgh, Pittsburgh, PA 15219, USA

**Keywords:** self-assembled monolayers, Ti-6Al-4V, carboxylic acids, diffuse reflectance infrared Fourier transform spectroscopy, matrix assisted laser desorption ionization-time of flight mass spectrometry, contact angle

## Abstract

The formation of methyl-terminated carboxylic acid self-assembled monolayers (SAMs) with even numbers of carbons, from eighteen to thirty, was investigated on the oxide surface of Ti-6Al-4V and component metal oxides. Modified surfaces were characterized using diffuse reflectance infrared Fourier transform spectroscopy (DRIFT), matrix assisted laser desorption ionization-time of flight mass spectrometry (MALDI-TOF MS) and contact angle analysis. Infrared spectroscopy indicated that using aerosol spray deposition techniques, stable, *all-trans* SAMs of octacosanoic (28 carbons) and triacontanoic (30 carbons) acids were formed on the alloy. Films were similarly formed on titanium and aluminum oxide. The surface of vanadium oxide exhibited limited reactivity. MALDI-TOF MS confirmed that formed films were monolayers, without multilayers or aggregates present. Water contact angles are indicative of the presence of hydrophobic methyl groups at the interface. This stable carboxylic acid SAM formation could be a useful alternative to phosphonic acid SAMs for corrosion and other applications.

## 1. Introduction

Titanium based alloys are widely used in engineering applications due to their high strength to weight ratio and corrosion resistance [1,2,3,4]. These mechanical characteristics have led to the widespread implementation of titanium alloys in applications ranging from biomedical devices to the aerospace industry [5,6,7,8]. Applications could be expanded if the properties of the oxide surface were changed through reaction with organic acid molecules to form self-assembled monolayers (SAMs) [6,9,10]. SAMs can be used to provide a chemically flexible interface. Changes to the chemistry of the interface include altered hydrophobicity, leading to improved corrosion resistance, and presentation of organic functional groups at the interface, which can be used in subsequent reactions resulting in immobilization of target compounds [11,12,13,14,15].

Prior work dealing with oxide surface modification of titanium alloys has focused mainly on the use of phosphonic acid molecules [10,16,17,18,19,20,21]. Phosphonic acid SAMs are well known and characterized, but the commercial availability of phosphonic acid molecules is limited compared to carboxylic acids, specifically pertaining to tail group functionality and various alkyl chain lengths [21,22,23,24]. There has been work studying the mechanism and kinetics of carboxylic acid film formation on TiO_2_ which has not yet been extrapolated to the alloy [25,26,27]. Therefore, developing routes for functionalizing Ti alloys with stable SAMs of carboxylic acid molecules, resulting in altered interfacial characteristics would be advantageous [28].

SAM formation and stability is a dynamic process that relies on interactions between the acid head group and the metal oxide surface, van der Waals interactions between chains and intermolecular forces between tail groups [24,29,30,31,32]. Limited surface reactivity or film stability may be able to be overcome by increasing other forces within the formed monolayer. These forces include van der Waals forces between adjacent alkyl chains, and tail group interactions, based on functionality, which may include hydrogen bonding or electrostatic forces [30,31,33].

In this study, carboxylic acid molecules with varying length carbon chains were examined for their ability to form *all-trans* alkyl chain monolayers on the oxide surface of Ti-6Al-4V, and component metal oxides, Ti, Al, and V. TLC aerosol spray deposition methods were used for SAM formation of octadecanoic (ODA), eicosanoic (ECA), docosanoic (DCA), tetracosanoic (TCA), hexacosanoic (HCA), octacosanoic (OCA) and triacontanoic (TAA) acids. Modified oxide surfaces were characterized by diffuse reflectance infrared Fourier transform spectroscopy (DRIFT), matrix assisted laser desorption ionization-time of flight mass spectrometry (MALDI-TOF MS) and contact angle analysis.

## 2. Results and Discussion

Self-assembled monolayer formation of carboxylic acids with even number chain lengths from 18–26 (ODA, ECA, DCA, TCA and HCA) was unsuccessful on the alloy. Several protocols for film formation were utilized with varied temperature and exposure time but these changes resulted in no film formation or disordered, incomplete surface coverage. An example protocol called for cooling the Ti-6Al-4V coupons on ice for an hour, followed by three cycles of TLC aerosol sprays using 1 mM acid solutions. Coupons were then heated in a 100 °C oven for 30 minutes, and the cycle was repeated three total times. Subsequent methods involved the same base protocol, but coupons were placed into a 120 °C oven, again resulting in no stable SAM formation. In subsequent protocols the exposure time was increased from three sprays to five sprays and the time in the oven between sprays was increased from 30 to 45 minutes. In separate protocols, the coupons were cooled using ice or dry ice and solution concentration was increased from 1 to 2 mM. However, these changes did not positively affect stable carboxylic acid SAM formation. Only changing the chain length to greater than 28 carbons (OCA and TAA) led to SAM formation on the alloy oxide using the initial protocol.

DRIFT spectroscopy was used to characterize the conformation of the alkyl chain and determine head group-oxide surface binding mode [23,33]. The positions of the methylene asymmetric and symmetric stretches are indicators of the order of the alkyl chains within the film [34,35,36,37]. An *all-trans* alkyl chain film will have methylene peaks with ν_CH2asymm _< 2918 cm^−1^ and ν_CH2symm _< 2850 cm^−1^, indicative of a crystalline organic solid [34,35,36,37]. Methylene peaks at wavenumbers greater than these indicate a liquid-like structure with *gauche* alkyl chain interactions [34,35]. The binding mode of the acid head group to the metal oxide surface is also examined through DRIFT [15,24,36]. Carboxylic acids have two potential binding modes, monodentate and bidentate. The binding mode is determined by comparing the head group region of the solid organic powder to that of the adhered molecules; changes between the spectra are indicative the binding mode.

**Figure 1:**
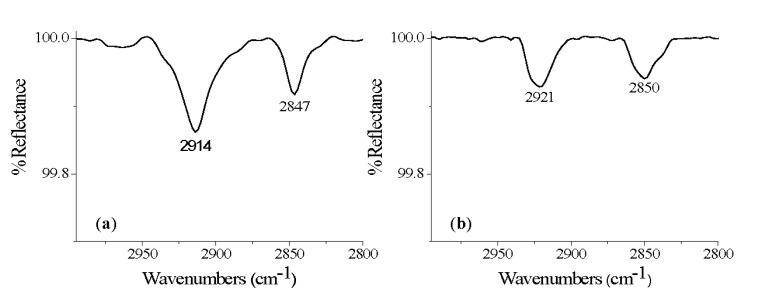
DRIFT spectra of methylene region of (**a**) hexacosanoic acid (HCA); and (**b**) eicosanoic acid (ECA) on Ti-6Al-4V, following sonication.

Acids containing 26 or less carbons in the molecule were found to form films that are either unstable or defective in some manner on the alloy. (For a complete summary of film formations on all substrates see Supplemental Information). These results are consistent with previous work on carboxylic acids on TiO_2_ which demonstrated that carboxylic acids with 16 carbons form monolayers on TiO_2_ but that the alkyl chains contained gauche interactions [26]. Hexacosanoic (HCA, 26) and tetracosanoic acids (TCA, 24) resulted in stable films, methylene peaks at ν_CH2asymm _= 2914 cm^−1^ and ν_CH2symm _= 2847 cm^−1^, but with incomplete surface coverage for HCA (Figure 1a) [17,38,39,40]. Modification using eicosanoic (ECA, 20) and docosanoic acid (DCA, 22) resulted in incomplete surface coverage and *gauche* alkyl chain interactions on Ti-6Al-4V, where ν_CH2asymm _= 2921 cm^−1^ and ν_CH2symm _= 2850 cm^−1^ for ECA (Figure 1b) [17,38,39,40]. For octadecanoic acid (ODA, 18), no protocols yielded films which were persistent through solvent sonication. This result may be attributable to smaller amounts of stabilizing forces within the SAM. The organic molecules with less than 26 carbons have less van der Waals interactions between adjacent carbon chains within the film. In this work, a decrease in van der Waals forces led to limited film stability through solvent sonication or prevented the formation of an *all-trans* alkyl chain film following reaction at the oxide surface which is consistent with previous work [41,42,43].

**Figure 2:**
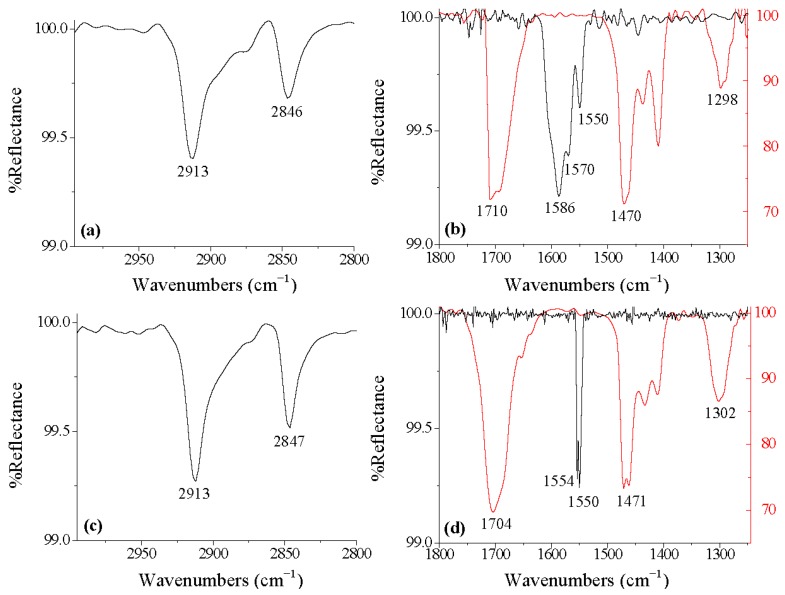
(**a**) DRIFT spectra of OCA methylene peaks; and (**b**) binding region after sonication; (**c**) DRIFT spectra of TAA methylene peaks; and (**d**) binding region after sonication. The solid acid powder is shown in red and the adhered acid is in black.

Ordered SAMs of OCA and TAA were successfully formed on the oxide surface of Ti-6Al-4V and component metal oxides surfaces using aerosol spray deposition methods. Coupons were cooled on ice for one hour, and then sprayed with 1 mM acid solutions. Following sprays, coupons were immediately transferred into a 100 °C oven. This process was repeated for a total of three cycles. DRIFT analysis of the methylene region of the formed OCA film on Ti-6Al-4V revealed peaks attributable to ν_CH2asymm _= 2913 cm^−1^ and ν_CH2symm _= 2846 cm^−1^, which are indicative of an *all-trans* alkyl chain film (Figure 2a). Further analysis of the C-O region of the spectra indicates that the OCA is interacting with the surface in a bidentate mode (Figure 2b). The DRIFT spectra of the solid OCA contained peaks attributed to ν_C=O_, ν_C-O_, and ν_C-OH_ at 1710, 1470 and 1298 cm^−1^, respectively, while the spectra of the adhered acid showed peaks attributed to ν_COO-_ at 1586, 1570 and 1550 cm^−1 ^(Figure 2b). Analysis of the TAA film revealed similar results, with ν_CH2asymm _= 2913 cm^−1^, ν_CH2symm _= 2847 cm^−1^ and ν_COO-_ at 1554 and 1550 cm^−1^ (Figure 2c,d). These DRIFT results indicate that TAA forms an *all-trans* alkyl chain film and the molecules are interacting with the surface in a bidentate mode. In each case the peaks are persistent through solvent rinse and sonication, indicating the film is stable and strongly adhered to the oxide surface.

Component metal oxide surfaces, Ti, Al, and V oxide, were modified independently to compare their reactivity to the titanium alloy’s reactivity. Modification of titanium, aluminum and vanadium oxide was performed with OCA and TAA using the same protocol described for the titanium alloy. DRIFT was again utilized to qualitatively assess the presence and conformation of the alkyl chain and determine the acid head group-oxide surface binding mode as shown in Table 1. Titanium and aluminum oxide modification with OCA yielded similar results to the titanium alloy. Methylene stretches indicated the presence of an *all-trans* alkyl chain OCA film where the molecules are interacting with the surface in a bidentate mode. However, none of the attempts to modify vanadium oxide resulted in stable SAMs, as the OCA was removed during solvent sonication in all cases.

Titanium and aluminum oxide modification with TAA resulted in films that were similar to those on the titanium alloy. DRIFT analysis of the methylene stretches revealed the presence of an *all-trans* alkyl chain film with the molecules interacting with the surface in a bidentate mode. Vanadium oxide modification with TAA resulted in a stable film that had *gauche* alkyl chain interactions and therefore a disordered surface film.

**Table 1:** Carboxylic Acid Modifications: Alkyl Chain Conformation and Binding Mode after Sonication.

Organic acid	Carbons in molecule	Oxide surface	-CH_2_- stretches (asymm, symm)	Binding mode (-dentate)
ODA	18	Ti-6Al-4V	-	-
ECA	20	Ti-6Al-4V	2921, 2850	-
DCA	22	Ti-6Al-4V	2922, 2850	-
TCA	24	Ti-6Al-4V	2918, 2848	-
HCA	26	Ti-6Al-4V	2914, 2847	-
OCA	28	Ti-6Al-4V	2913, 2846	Bi
-	-	Ti	2914, 2847	Bi
-	-	Al	2914, 2848	Bi
-	-	V	-	-
TAA	30	Ti-6Al-4V	2913, 2847	Bi
-	-	Ti	2912, 2847	Bi
-	-	Al	2917, 2849	Bi
-	-	V	2923, 2852	Bi

**Figure 3:**
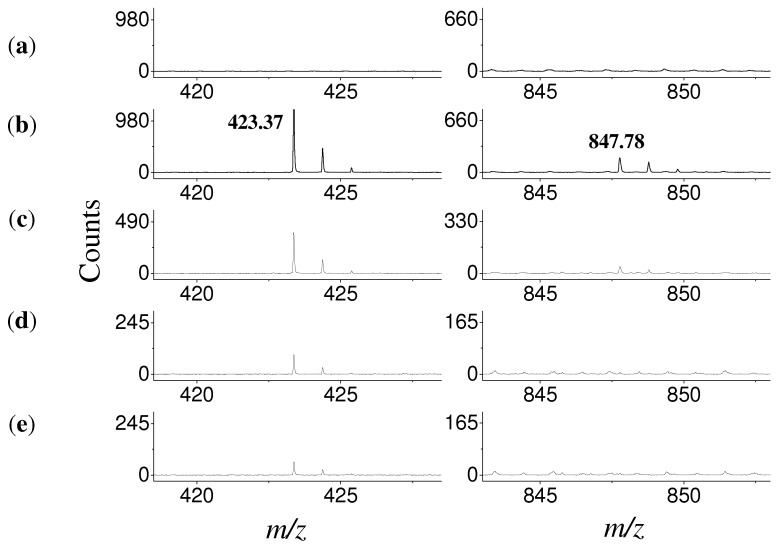
MALDI-TOF mass spectra of octacosanoic acid deposited onto Ti-6Al-4V following each step of SAM formation. The monomer region is shown in the left column and the dimer region in the right column. (**a**) *All-trans* retinoic acid matrix; (**b**) Solid octacosanoic acid; both the monomer (1168 counts) and the dimer (186 counts) can be detected via MALDI-TOF MS; (**c**) Octacosanoic acid film following aerosol spray deposition (monomer 391 counts; dimer 43 counts); (**d**) Octacosanoic acid SAM following solvent (THF) rinse (monomer 94 counts; dimer 0); (**e**) Octacosanoic acid SAM following solvent sonication (monomer 63 counts; dimer 0); Peaks detected in the dimer range of the mass spectra in (**a**), (**d**) and (**e**) are attributed to noise from the MALDI-TOF MS with peak heights < 2 counts.

Quantitative assessment of film thickness was carried out using MALDI-TOF MS to distinguish between mono- and multilayer coverage [44]. MALDI-TOF MS analysis of the OCA and TAA was performed using negative ionization mode. Prior to collecting mass spectra of modified coupons, spectra of *all-trans* retinoic acid matrix (Figure 3a) and solid acid powder (Figure 3b) were collected to ensure no matrix peaks would interfere with SAM analysis. It was clear from the spectra that no matrix interference was present and the location of the peaks corresponding to the acid monomer and dimer were determined (Figure 3b). The peaks relating to the monomer of [OCA-H]^-^ and [TAA-H]^-^ can be detected at 423.37 *m/z* and 451.44 *m/z* respectively. If the dimer peaks were present in the mass spectra, corresponding to [2 OCA-H]^-^ at 847.78 *m/z* and [2 TAA-H]^-^ at 903.92 *m/z*, it would indicate that the present film consists of a multilayer of molecules (Figure 3b). MALDI-TOF MS was carried out following each step of the SAM formation process, including deposition (Figure 3c), solvent rinse (Figure 3d) and solvent sonication (Figure 3e). Following deposition, MALDI-TOF MS indicated the presence of OCA multilayers by detection of both monomeric and dimeric peaks. The transition from multilayers to SAMs was seen following the solvent rinse and sonication procedures, where only the monomer of OCA was detected.

Upon determining SAMs to be present in an *all-trans* conformation, contact angle analysis of the coupons modified with the carboxylic acids was used to determine the wetting properties of the coupons. The formed SAMs were methyl terminated and therefore should exhibit increased hydrophobicity when compared to the unmodified, control Ti-6Al-4V coupons. Collected contact angle data showed that the formed OCA and TAA films presented a more hydrophobic interface when compared to the control Ti-6Al-4V. The contact angle for control Ti-6Al-4V was found to be 78° ± 2°, this value is comparable to previously reported contact angles of metal oxides [15,22,23]. The methyl-terminated OCA and TAA films had increased contact angles compared to the control of 106° ± 2° and 102° ± 1°, respectively; consistent with previously reported methyl-terminated SAMs on metal oxide surfaces [37,45,46].

Modification of the oxide surface of the titanium alloy, Ti-6Al-4V, resulted in the formation of stable self-assembled monolayers. Diffuse reflectance infrared Fourier transform spectroscopy indicated that octacosanoic and triacontanoic acid films were present in an *all-trans* confirmation and stable through solvent sonication. Component metal oxide modification resulted in *all-trans* SAM formation on titanium and aluminum oxide, whereas SAM formation was limited on vanadium oxide. Additionally, shorter chain carboxylic acids, with an even number of carbon atoms between octadecanoic and hexacosanoic acid, showed a limited ability to form stable films on the titanium alloy’s oxide surface. Matrix assisted laser desorption ionization-time of flight mass spectrometry and contact angle analysis further confirmed the formation of hydrophobic monolayer films on the alloy coupons.

## 3. Experimental

### 3.1. Materials

Octadecanoic acid (ODA, 95%), eicosanoic acid (ECA, 99%), docosanoic acid (DCA, 99%) and octacosanoic acid (OCA, 98%) were purchased from Sigma-Aldrich (St. Louis, MO, USA). Tetracosanoic acid (TCA, 96%), hexacosanoic acid (HCA, 95%) and triacontanoic acid (TAA, 95%) were purchased from TCI America (Portland, OR, USA). *All-trans* retinoic acid (TRA, 98%), used in mass spectrometry analysis (MALDI-TOF MS), was purchased from Alfa Aesar (Ward Hill, MA, USA), and all reagents were used as received. Tetrahydrofuran (THF, Optima grade) was purchased from Fischer Scientific (Rockingham, NH, USA) and distilled over sodium and benzophenone before use. Titanium alloy (Ti-6Al-4V, 99.9%), titanium (Ti, 99.6%), and aluminum (Al, 99.0%) foils of 0.25 mm thickness were purchased from Goodfellow, Inc. (Delson, QC, Canada), and 0.25 mm thick vanadium (V, 99.7%) foil was purchased from Sigma-Aldrich (St. Louis, MO, USA). All foils were prepared by the protocol described in Section 3.2.

### 3.2. Coupon Preparation

Ti-6Al-4V, Ti, Al, and V foils are prepared by sanding with 150, 320, 400, and 600 grit sandpaper sequentially, then cutting the foils into 1 cm x 1 cm coupons. Sanding of coupons has previously been shown to result in surface elemental weight percentages that are different than bulk elemental weight percentages. A study on Ti-6Al-4V found that the average surface elemental weight percent exhibited was decreased for titanium (86 ± 6%) and vanadium (2 ± 1%) with an increased percent of aluminum (12 ± 2%) [47]. After sanding coupons are cleaned by rinse and sonication in acetone for 30 minutes, followed by immersion in boiling methanol for 15 minutes. This process removes residual metallic dust and organic residue from the metal oxide coupons. Clean coupons are stored at 100 °C until use.

### 3.3. SAM Formation Protocols

SAMs were formed using a TLC aerosol sprayer with nitrogen stream deposition technique, where the organic acid solutions, in dry THF, are sprayed onto the metal oxide coupons [24,44]. A series of increasingly aggressive protocols which varied by temperature, acid concentration, number of spray coatings and coupon drying conditions was attempted with each acid in an attempt to optimize SAM formation. Table 2 outlines the initial procedures used which produced no film formation for any of the acids. Protocols outlined in section 3.5 led to SAM formation. While the final conditions for successful SAM formation vary by chain length, each protocol was attempted for each chain length and varied as necessary.

### 3.4. Protocols Not Resulting in SAM Formation

#### Octadecanoic (18 Carbons) Acid

All of the protocols described herein were used with octadecanoic acid. However, no protocols resulted in film formation on any of the coupons.

For example protocols not resulting in SAM formation, please see Table 2.

**Table 2:** Additional example protocols not resulting in SAM formation.

Protocol	Organic acid	Method used	Solution conc. (mM)	Cooling method	Sprays	Solvent removal	Oven time (min)	Oven temp. (°C)
1	ODA-TAA	TLC Spray	1	Ice, 1 hr.	3	Oven	30	100
2	ODA-HCA	TLC Spray	1	Ice, 1 hr.	3	Oven	30	120
3	ODA-HCA	TLC Spray	1	Ice, 1 hr.	5	Oven	45	100
4	ODA-HCA	TLC Spray	1	Ice, 1 hr.	3	Ambient	30	100
5	ODA-HCA	TLC Spray	1	Ice, 1 hr.	5	0.1 torr vac line	30	100
6	ODA-HCA	Solution (50°C, 3hr)	2	Ice, 1 hr.	-	Oven	-	100

### 3.5. Protocols Resulting in SAM Formation

#### 3.5.1. Eicosanoic (20 Carbons) Acid

Eicosanoic acid films were formed using a TLC spray deposition method. The coupons were first cooled on ice in a glass dish for one hour then sprayed with a 1 mM acid solution. Coupons were immediately transferred to a 100 °C oven for 45 minutes. Coupons were placed back on ice for 20 minutes and cooled before the next spray. This procedure was repeated an additional four times.

#### 3.5.2. Docosanoic (22 Carbons) Acid

Docosanoic acid films were formed using a TLC aerosol spray deposition method. Ti-6Al-4V coupons were placed in a glass dish on ice for one hour. A 1 mM acid solution was sprayed onto the coupons, and solvent is removed at ambient conditions. After solvent removal coupons were placed into a 100 °C oven for 30 minutes. Once removed from the oven, coupons were cooled for 20 minutes. The procedure was repeated two additional times.

#### 3.5.3. Tetracosanoic (24 Carbons) and Hexacosanoic (26 Carbons) Acids

Tetracosanoic and hexacosanoic films were formed using a solution deposition method. Cleaned coupons were cooled in a glass dish in an acetone/dry ice bath for 30 minutes. Following cooling the coupons were placed into a 2 mM solution of the respective organic acid for 15 minutes. Solvent was then evaporated at ambient conditions. Following solvent removal the coupons were stored in the 100 °C oven.

#### 3.5.4. Octacosanoic (28 Carbons) and Triacontanoic (30 Carbons) Acids

Octacosanoic and triacontanoic acid films were formed using a TLC aerosol spray deposition method. Cleaned coupons were cooled in a glass dish on ice for one hour, and then a 1 mM acid solution was sprayed onto the coupons. Following the spraying, coupons were immediately transferred to the 100 °C oven. Coupons remained in the oven for 30 minutes. Following the 30 minutes, coupons were removed and placed back on ice for 20 minutes. This entire procedure was repeated two additional times, for three total cycles.

### 3.6. Infrared Spectroscopy

Diffuse reflectance infrared Fourier transform (DRIFT) spectroscopy was used to examine the alkyl chain methylene stretches, which confirm the presence of the organic acid molecule. A Nexus 470 Fourier transform infrared spectrometer with DRIFT attachment analyzes the carbon chain and acid head group regions of the adhered carboxylic acid molecules. All DRIFT analysis was performed under nitrogen purge for 256 scans at 4 cm^−1^ resolution.

### 3.7. Mass Spectrometry

Matrix-assisted laser desorption ionization-time of flight mass spectrometry (MALDI-TOF MS), using a 10 mg/mL *all-trans* retinoic acid (TRA) matrix solution in THF, was used to distinguish between monolayers and multilayers of molecules on the oxide surfaces [44]. An Agilent Technologies G1969A LC/MSD TOF mass spectrometry system with G1974A AP/MALDI ion source attachment in negative ionization mode was used. Mass spectra were collected for 2.0 minutes with N_2_ gas flow of 10 L/min at a temperature of 325 °C, a capillary voltage of 3,200 V, fragmentor voltage of 220 V and skimmer voltage of 50 V. MALDI-TOF MS samples were prepared using the dried-droplet method, where 1 μL of matrix solution is dropped onto the modified coupons and dried prior to analysis [44,48]. To obtain a representative mass spectrum of each modification, the laser was used for ablation of three spots on each coupon.

### 3.8. Contact Angle Analysis

Contact angle analysis was used to evaluate the wetting properties of the carboxylic acid SAMs formed on Ti-6Al-4V. In contact angle analysis, a Video Contact Angle (VCA) 2000 instrument measured the left and right advancing contact angles (θ) of 18 mΩ deionized water. A syringe deposited 1 μL of deionized water on the coupon for analysis. Contact angles were acquired in three different locations on three samples. A one way analysis of variance (ANOVA) with a Bonferroni post-hoc test with *p* < 0.05, was used to compare the contact angles for the control and modified coupons.

### 3.9. SAM Stability Assessment

Solvent rinse and sonication were performed in dry tetrahydrofuran for 15 and 20 minutes, respectively. Solvent rinse was carried out by coupon immersion at ambient conditions without external agitation. Sonication was done with a Branson Ultrasonic Cleaner, model 1510R-MT with 42 kHz and 70 watt output capability. Solvent rinse and sonication served to remove any physisorbed organic acid molecules as well as assessed acid molecule stability on the metal oxide surface.

## 4. Conclusions

Stable, *all-trans* alkyl chain monolayers of octacosanoic and triacontanoic acids, 28 and 30 carbons respectively, were formed on the oxide surface of the titanium alloy, Ti-6Al-4V, as well as on the component metal oxides of titanium and aluminum. DRIFT analysis confirmed that the formed films were *all-trans* and stable through solvent rinse and sonication. MALDI-TOF MS and contact angle analysis further confirmed that films were present as monolayers and the interfaces are hydrophobic. Carboxylic acids with even numbers of carbons in the alkyl chain between 18 and 26 were also used for film formation. However, disordered, unstable films resulted. These results are indicative of the importance of the role the van der Waals forces play in carboxylic acid film formation and stability on the titanium alloy and its component metal oxides.

## Supplementary Materials

Supplementary File 1
