# Supplementary material for: Effect of Alkyl Chain Length on Carboxylic Acid SAMs on Ti-6Al-4V
Source: Materials (Basel). 2012 Jul 9;5(7):1206–18. doi: 10.3390/ma5071206 (PMC5448898; doi:10.3390/ma5071206)
Supplement: Supplementary File 1 [file materials-05-01206-s001.pdf]

Supplementary Information

Monolayer Formation

Using the conditions delineated in the Experimental section of the manuscript, film formation was attempted on the Ti-6Al-4V and its component oxides. In the subsequent tables, the resulting CH_2 symmetric and asymmetric values for each sample, as determined by DRIFT spectroscopy, can be found after deposition and sonication. Only substrates with *all-trans* alkyl chains through sonication are considered to be ordered, stable films. An *all-trans* alkyl chain film will have methylene peaks with $\nu_{CH2asymm} < 2918\ cm^{-1}$ and $\nu_{CH2symm} < 2850\ cm^{-1}$. Methylene peaks at wavenumbers greater than these indicate a film with a liquid-like structure with *gauche* alkyl chain interactions. Table S1 summarizes the protocols used in attempted film formation on the oxide surfaces. Tables S2 and S3 contain the results obtained using Protocol 1 for triacontanoic and octacosanoic acid, respectively. Tables S4–S8 show the results obtained for hexacosanoic, tetracosanoic, docosanoic, eicosanoic and octadecanoic acids, respectively, using the six protocols described here.

Protocol 1

Protocol 1 utilized a TLC aerosol spray deposition method. Cleaned coupons were cooled in a glass dish on ice for one hour, and then a 1 mM acid solution was sprayed onto the coupons. Following the spraying, coupons were immediately transferred to the 100 °C oven. Coupons remained in the oven for 30 minutes. Following the 30 minutes, coupons were removed and placed back on ice for 20 minutes. This entire procedure was repeated two additional times, for three total cycles.

Protocol 2

Protocol 2 utilized a TLC aerosol spray deposition method. Cleaned coupons were cooled in a glass dish on ice for one hour, and then a 1 mM acid solution was sprayed onto the coupons. Following the spraying, coupons were immediately transferred to the 120 °C oven. Coupons remained in the oven for 30 minutes. Following the 30 minutes, coupons were removed and placed back on ice for 20 minutes. This entire procedure was repeated two additional times, for three total cycles.

Protocol 3

Protocol 3 utilized a TLC aerosol spray deposition method. The coupons were first cooled on ice in a glass dish for one hour then sprayed with a 1 mM acid solution. Coupons were immediately transferred to a 100 °C oven for 45 minutes. Coupons were placed back on ice for 20 minutes and cooled before the next spray. This procedure was repeated an additional four times.

Protocol 4

Protocol 4 utilized a TLC aerosol spray deposition method. Ti-6Al-4V coupons were placed in a glass dish on ice for one hour. A 1 mM acid solution was sprayed onto the coupons, and solvent is removed at ambient conditions. After solvent removal coupons were placed into a 100 °C oven for 30 minutes.

Once removed from the oven, coupons were cooled for 20 minutes. The procedure was repeated two additional times.

Protocol 5

Protocol 5 utilized a TLC aerosol spray deposition method. Ti-6Al-4V coupons were placed in a glass dish on ice for one hour. A 1 mM acid solution was sprayed onto the coupons, and solvent is removed using a 0.1 torr vacuum line. After solvent removal coupons were placed into a 100 °C oven for 30 minutes. Once removed from the oven, coupons were cooled for 20 minutes. The procedure was repeated four additional times.

Protocol 6

Protocol 6 utilized a solution deposition method. Ti-6Al-4V coupons were placed in a glass dish on ice for one hour. A 2 mM acid solution was heated to 50 °C and cold coupons were immersed for 3 hours. After 3 hours the coupons were removed and placed into a 100 °C oven.

Table S1. Protocols Attempted on the oxide surfaces of Ti-6Al-4V, Ti, Al and V.

Protocol	Organic acid	Method used	Solution conc. (mM)	Cooling method	Sprays	Solvent removal	Oven time (min)	Oven temp. (°C)
1	ODA-TAA	TLC Spray	1	Ice, 1 hr.	3	Oven	30	100
2	ODA-HCA	TLC Spray	1	Ice, 1 hr.	3	Oven	30	120
3	ODA-HCA	TLC Spray	1	Ice, 1 hr.	5	Oven	45	100
4	ODA-HCA	TLC Spray	1	Ice, 1 hr.	3	Ambient	30	100
5	ODA-HCA	TLC Spray	1	Ice, 1 hr.	5	0.1 torr vac line	30	100
6	ODA-HCA	Solution (50°C, 3hr)	2	Ice, 1 hr.	-	Oven	-	100

Table S2. Methylene stretches of triacontanoic acid on the oxide surfaces of Ti-6Al-4V, Ti, Al and V following deposition and solvent sonication.

Protocol	Triacontanoic acid (30 Carbons)	Methylene stretches ($\nu_{CH2\ asymm}$, $\nu_{CH2\ symm}$)	
	Oxide surface	Deposition	Sonication
1	Ti-6Al-4V	2912, 2847	2913, 2847
	Ti	2913, 2846	2912, 2847
	Al	2914, 2847	2917, 2849
	V	2914, 2847	2923, 2852

Table S3. Methylene stretches of octacosanoic acid on the oxide surfaces of Ti-6Al-4V, Ti, Al and V following deposition and solvent sonication.

Protocol	Octacosanoic acid (28 Carbons)	Methylene stretches ($\nu_{CH2\ asymm}$, $\nu_{CH2\ symm}$)	
	**Oxide surface**	**Deposition**	**Sonication**
1	Ti-6Al-4V	2914, 2847	2913, 2846
	Ti	2914, 2847	2914, 2847
	Al	2914, 2849	2914, 2848
	V	2916, 2848	-

Table S4. Methylene stretches of hexacosanoic acid on the oxide surfaces of Ti-6Al-4V, Ti, Al and V following deposition and solvent sonication.

Protocol	Hexacosanoic acid (26 Carbons)	Methylene stretches ($\nu_{CH2\ asymm}$, $\nu_{CH2\ symm}$)	
	**Oxide surface**	**Deposition**	**Sonication**
1	Ti-6Al-4V	2914, 2846	2922, 2952
	Ti	2914, 2846	2913, 2846
	Al	2913, 2846	2915, 2849
	V	-	-
2	Ti-6Al-4V	-	-
	Ti	2918, 2849	-
	Al	2921, 2850	2922, 2851
	V	-	-
3	Ti-6Al-4V	2914, 2846	-
	Ti	2913, 2846	-
	Al	2912, 2846	2916, 2848
	V	-	-
4	Ti-6Al-4V	2916, 2848	-
	Ti	2913, 2846	-
	Al	2914, 2847	2914, 2848
	V	-	-
5	Ti-6Al-4V	2915, 2846	-
	Ti	2915, 2847	-
	Al	2914, 2848	2914, 2848
	V	-	-
6	Ti-6Al-4V	-	-
	Ti	-	-
	Al	-	-
	V	-	-

Table S5. Methylene stretches of tetracosanoic acid on the oxide surfaces of Ti-6Al-4V, Ti, Al and V following deposition and solvent sonication.

Protocol	Tetracosanoic acid (24 Carbons)	Methylene stretches ($\nu_{CH2\ asymm}$, $\nu_{CH2\ symm}$)	
	Oxide surface	Deposition	Sonication
1	Ti-6Al-4V	2915, 2847	-
	Ti	2916, 2848	-
	Al	2915, 2847	2915, 2847
	V	-	-
2	Ti-6Al-4V	-	-
	Ti	-	-
	Al	-	-
	V	-	-
3	Ti-6Al-4V	2915, 2846	-
	Ti	2915, 2847	-
	Al	2923, 2853	2922, 2852
	V	-	-
4	Ti-6Al-4V	2915, 2847	-
	Ti	-	-
4	Al	2915, 2848	-
	V	-	-
5	Ti-6Al-4V	2916, 2848	-
	Ti	-	-
	Al	2915, 2848	2914, 2845
	V	-	-
6	Ti-6Al-4V	-	-
	Ti	-	-
	Al	-	-
	V	-	-

Table S6. Methylene stretches of docosanoic acid on the oxide surfaces of Ti-6Al-4V, Ti, Al and V following deposition and solvent sonication.

Protocol	Docosanoic acid (22 Carbons)	Methylene stretches ($\nu_{CH2\ asymm}$, $\nu_{CH2\ symm}$)	
	**Oxide surface**	**Deposition**	**Sonication**
1	Ti-6Al-4V	2918, 2850	-
	Ti	2914, 2847	-
	Al	2915, 2848	2915, 2848
	V	-	-
2	Ti-6Al-4V	-	-
	Ti	2917, 2848	-
	Al	-	-
	V	-	-
3	Ti-6Al-4V	2918, 2848	2922, 2850
	Ti	2915, 2847	-
	Al	2914, 2847	2915, 2849
	V	-	-
4	Ti-6Al-4V	2917, 2848	2922, 2850
	Ti	2916, 2848	-
	Al	2914, 2848	2915, 2848
	V	-	-
5	Ti-6Al-4V	2914, 2847	-
	Ti	2917, 2848	-
	Al	2916, 2849	2916, 2849
	V	-	-
6	Ti-6Al-4V	-	-
	Ti	-	-
	Al	-	-
	V	-	-

Table S7. Methylene stretches of eicosanoic acid on the oxide surfaces of Ti-6Al-4V, Ti, Al and V following deposition and solvent sonication.

**Protocol**	**Eicosanoic acid (20 Carbons)**	**Methylene stretches ($\nu_{CH2\ asymm}$, $\nu_{CH2\ symm}$)**	
	**Oxide surface**	**Deposition**	**Sonication**
1	Ti-6Al-4V	2914, 2847	-
	Ti	2916, 2847	-
	Al	2915, 2848	2923, 2851
	V	-	-
2	Ti-6Al-4V	2915, 2847	-
	Ti	-	-
	Al	2922, 2851	-
	V	-	-
3	Ti-6Al-4V	2914, 2847	2921, 2850
	Ti	2916, 2848	-
	Al	2922, 2851	2921, 2851
	V	-	-
4	Ti-6Al-4V	2921, 2851	-
	Ti	-	-
	Al	2914, 2848	-
	V	-	-
5	Ti-6Al-4V	2914, 2846	-
	Ti	2917, 2850	-
	Al	2916, 2849	2915, 2848
	V	-	-
6	Ti-6Al-4V	-	-
	Ti	-	-
	Al	-	-
	V	-	-

Table S8. Methylene stretches of octadecanoic acid on the oxide surfaces of Ti-6Al-4V, Ti, Al and V following deposition and solvent sonication.

Protocol	Octadecanoic acid (18 Carbons)	Methylene stretches ($\nu_{CH2\ asymm}$, $\nu_{CH2\ symm}$)	
	Oxide surface	Deposition	Sonication
1	Ti-6Al-4V	2917, 2849	-
	Ti	2915, 2847	-
	Al	2915, 2847	2917, 2849
	V	-	-
2	Ti-6Al-4V	2919, 2850	-
	Ti	2915, 2846	-
	Al	2918, 2850	-
	V	-	-
3	Ti-6Al-4V	2916, 2848	-
	Ti	2914, 2846	-
	Al	2916, 2848	2917, 2849
	V	-	-
4	Ti-6Al-4V	2915, 2845	-
	Ti	2914, 2845	-
	Al	2916, 2847	2915, 2847
	V	-	-
5	Ti-6Al-4V	-	-
	Ti	2916, 2846	-
	Al	2918, 2849	2916, 2847
	V	-	-
6	Ti-6Al-4V	-	-
	Ti	-	-
	Al	-	-
	V	-	-

© 2012 by the authors; licensee MDPI, Basel, Switzerland. This article is an open access article distributed under the terms and conditions of the Creative Commons Attribution license (http://creativecommons.org/licenses/by/3.0/).
